# MiR34a Regulates Neuronal MHC Class I Molecules and Promotes Primary Hippocampal Neuron Dendritic Growth and Branching

**DOI:** 10.3389/fncel.2020.573208

**Published:** 2020-10-28

**Authors:** Yue Hu, Wenqin Pei, Ying Hu, Ping Li, Chen Sun, Jiawei Du, Ying Zhang, Fengqin Miao, Aifeng Zhang, Yuqing Shen, Jianqiong Zhang

**Affiliations:** ^1^Department of Microbiology and Immunology, Key Laboratory of Developmental Genes and Human Disease, Ministry of Education, Medical School, Southeast University, Nanjing, China; ^2^Department of Pathology, Medical School, Southeast University, Nanjing, China; ^3^Jiangsu Key Laboratory of Molecular and Functional Imaging, Zhongda Hospital, Medical School, Southeast University, Nanjing, China

**Keywords:** hippocampal development, dendritic growth and branching, primary hippocampal neurons, miR-34a, MHC class I

## Abstract

In the immune system, Major Histocompatibility Complex class I (MHC-I) molecules are located on the surface of most nucleated cells in vertebrates where they mediate immune responses. Accumulating evidence indicates that MHC-I molecules are also expressed in the central nervous system (CNS) where they play important roles that are significantly different from their immune functions. Classical MHC-I molecules are temporally and spatially expressed in the developing and adult CNS, where they participate in the synaptic formation, remodeling and plasticity. Therefore, clarifying the regulation of MHC-I expression is necessary to develop an accurate understanding of its function in the CNS. Here, we show that microRNA 34a (miR34a), a brain enriched noncoding RNA, is temporally expressed in developing hippocampal neurons, and its expression is significantly increased after MHC-I protein abundance is decreased in the hippocampus. Computational algorithms identify putative miR34a target sites in the 3′UTR of MHC-I mRNA, and here we demonstrate direct targeting of miR34a to MHC-I mRNA using a dual-luciferase reporter assay system. MiR34a targeting can decrease constitutive MHC-I expression in both Neuro-2a neuroblastoma cells and primary hippocampal neurons. Finally, miR34a mediated reduction of MHC-I results in increased dendritic growth and branching in cultured hippocampal neurons. Taken together, our findings identify miR34a as a novel regulator of MHC-I for shaping neural morphology in developing hippocampal neurons.

## Introduction

For a long time, the central nervous system (CNS) has been considered to be “immune-privileged.” This view, however, has changed following the identification of classical immune molecules such as cytokines, complement, and major histocompatibility complex (MHC) proteins in the healthy, uninfected CNS (Joly et al., 1991). Several studies have demonstrated that these immune proteins impart pleiotropic effects on neurons (Boulanger et al., 2001). In the immune system, classical MHC-I proteins are located on the surface of most nucleated cells where they regulate T cell and NK cell-mediated immune responses. In the vertebrate CNS, MHC-I proteins are constitutively expressed on the surface of axons and dendrites of some neurons over discrete developmental and adult stages (Elmer and Mcallister, 2012). Decades of research has now definitively shown that classical MHC-I molecules play extremely important and diverse roles in the CNS including regulation of synapse formation, remodeling, and plasticity and that these roles are distinct from their originally identified immune functions (Corriveau et al., 1998; Boulanger, 2009; Elmer and Mcallister, 2012).

Our previous studies reported on the specific spatial and temporal expression pattern of classical MHC-I molecules in developing C57BL/6 mouse brains. In the developing cerebellum and hippocampus, levels of MHC-I protein gradually increase from postnatal day 0 (P0) to a peak value at P15, a crucial period for synaptic remodeling and plasticity. After completing their functions, protein abundance becomes significantly reduced with age until neuronal MHC-I proteins can no longer be detected after P60. In contrast, mRNA expression is gradually increased from P0 to P60 after which it remains relatively stable (Liu et al., 2013; Li et al., 2020). A similar expression pattern of MHC-I proteins is observed during human CNS development (Zhang et al., 2013a,b). This evidence suggests that the expression of MHC-I proteins is tightly regulated in the developing CNS due to their role in important neurodevelopmental events. Due to this discrepancy between protein abundance and mRNA levels during development, the complex regulation of MHC-I expression at the transcriptional and post-transcriptional level needs to be further investigated.

We recently found that NLRC5 is a critical transcriptional factor of MHC-I during development from P0 to P15 in hippocampal neurons (Li et al., 2020). All previous studies on the post-transcriptional regulation of MHC-I expression have only detailed regulation in the immune system. MEX-3C acts as a novel ubiquitin E3 ligase, binding to the 3′UTR of HLA-A2 mRNA and degrading it to fine-tune regulation of HLA-A protein abundance (Cano et al., 2012). RNA-binding protein HNRNPR positively regulates the expression of both classical and nonclassical MHC-I proteins through binding to the 3′UTR of HLA-A, -B, -C, and -G mRNA and enhancing protein stability (Reches et al., 2016). MicroRNA hsa-miR148 is increased in HIV infected cells to decrease HLA-C expression and evade host immune recognition (Kulkarni et al., 2011, 2013). Although we know some transcriptional factors can regulate MHC-I expression at the level of transcription in the developing CNS, whether it is regulated post-transcriptionally remains poorly understood.

MicroRNAs (miRNAs) are small non-coding RNAs. They function as post-transcriptional regulators and target the 3′UTR of messenger RNA transcripts (mRNAs). MiRNA binding can either result in the degradation of genetic transcripts or can result in silencing mRNA translation indirectly (Bartel, 2009). Accumulating evidence demonstrates microRNAs play a non-negligible role in modulating neuronal activities and maintaining homeostasis of the CNS (Davis et al., 2015; Yu et al., 2015; Evgenia and De Strooper, 2017). Our study reveals miR34a functions as a negative regulator of classical MHC-I molecules in both Neuro-2a cells and in primary cultured mouse hippocampal neurons. In mice, miR34a is especially abundant in brain tissue and participates in neuronal morphology and function by modulating the expression of synaptic targets (Agostini et al., 2011a,b; Morgado et al., 2015). However, its relationship with MHC-I has not yet been reported. We find that the expression of miR34a is negatively correlated with that of MHC-I protein levels in the developing hippocampus. We also demonstrate that the putative miR34a target sites are located in MHC-I mRNA 3′UTR. By binding to the 3′UTR region of MHC-I mRNA, miRNA-34a decreases the level of MHC-I mRNA and proteins. Inhibition of miR34a decreases dendritic growth and branching in cultured hippocampal neurons due to enhanced MHC-I protein expression. All these findings provide the first evidence that miRNA-34a acts as a novel regulator in modulating neuronal MHC-I.

## Materials and Methods

### Animals

Experimental animals were C57BL/6J mice (Cat# n000013c57, RRID: MGI:5657312) purchased from GemPharmatech Company, Limited (China) in this study. The animals were housed in polypropylene cages under pathogen-free conditions of constant temperature and humidity, maintained on a 12–12 h day-night cycle, with food and water provided *ad libitum* in the standard animal facility. Animal experiments were conducted according to the protocols evaluated and approved by the Institutional Animal Care and Use Committee (IACUC) of the Medical School of Southeast University (approval ID: SYXK-2010.4987). Postnatal day P0 was designated as the day of birth. P0, P8, P15, P30, and P60 male mice were used for experiments. Fresh tissues, RNA, protein, or others extracted from mice were stored in liquid nitrogen.

### Cell Lines

Mouse neuroblastoma N2a cells, also known as Neuro-2a cells (RRID: CVCL_0470), are murine neuroblastoma cells obtained from the cell bank of the Type Culture Collection of the Chinese Academy of Science (China). Cell lines employed in our laboratory were authenticated in 2019 using short tandem repeat (STR) analysis at Shanghai Biowing Biotechnology Company, Limited. Neuro-2a cells were seeded in 25-cm^2^ cell culture flasks in modified Eagle’s medium (MEM; Gibco, USA) with an additional 10% FBS (Gibco-BRL, USA) and 1% penicillin-streptomycin antibiotics (Gibco-BRL, USA). The growth medium was changed every 2–3 days. Before each experiment, monolayers of cells were trypsinized and diluted in MEM, then transferred to new culture plates. Neuro-2a cells were transfected with miR34a mimic or miR34a inhibitor (Ribo Life Science, China). The following microRNA mimics and inhibitors were used: miR34a mimic upstream sequence: 5′-UGGCAGUGUCUUAGCUGGUUGU-3′, and downstream sequence: 5′-AACCAGCUAAGACACUGCCAUU-3′; miR34a inhibitor: 5′-ACAACCAGCUAAGACACUGCCA-3′; mimic negative control upstream sequence: 5′-UUCUUCGAACGUGUCACGUTT-3′, and downstream sequence: 5′-ACGUGACACGUUCGGAGAATT-3′; inhibitor negative control upstream sequence: 5′-CAGUACUUUUGUGUAGUACAA-3′. To ensure the characteristics of Neuro-2a cells, cells used in all the experiments were not passaged for more than 10 times.

### Primary Neuron Culture

Hippocampal neuronal cells were isolated from E16.5 to 18.5 fetus as previously described (Kaech and Banker, 2006). Primary hippocampal neurons were seeded in 6-well plates, or on glass coverslips (coated with 100 μg/ml poly-d-lysine, Sigma–Aldrich, USA) placed in 24-well plates. Cells were cultured in Neurobasal media (Gibco, USA) supplemented with GlutaMAX (Invitrogen, USA) and glucose (Sigma–Aldrich, USA). An optimized serum-free supplement B27 (Invitrogen, USA) was added to the Neurobasal Medium. The growth medium was changed after 3 DIV by half volume. The lentivirus used in this study were synthesized by Shanghai Genechem Company, Limited. Gene sequences inserted in lentivirus are shown in Supplementary Material and Methods.

### Luciferase Reporter Assay

The full length of H-2D^b^ and H-2K^b^ 3′UTR containing the putative miRNA target sites and the mutated sequence was chemically synthesized and cloned into pmiRGLO vector (Genewiz, China). The sequences of H-2D^b^ and H-2K^b^ 3′UTR are shown in Supplementary Material and Methods. HET293T cells were transfected with the H-2D^b^/H-2K^b^ 3′UTR pmiRGLO vector, or mutated H-2D^b^/H-2K^b^ 3′UTR pmiRGLO vector and microRNA mimics (Ribo Life Science, China). The following microRNA mimics were used in this study: miR34a mimic upstream sequence: 5′-UGGCAGUGUCUUAGCUGGUUGU-3′, and downstream sequence: 5′-AACCAGCUAAGACACUGCCAUU-3′; miR146b mimic upstream sequence: 5′-UGAGAACUGAAUUCCAUAGGCU-3′, and downstream sequence: 5′-CCUAUGGAAUUCAGUUCUCAUU-3′; miR148a mimic upstream sequence: 5′-UCAGUGCACUACAGAACUUUGU-3′, and downstream sequence: 5′-AAAGUUCUGUAGUGCACUGAUU; miR181a mimic upstream sequence: 5′-AACAUUCAACGCUGUCGGUGAGU-3′, and downstream sequence: 5′-UCACCGACAGCGUUGAAUGUUUU-3′; miR181c mimic upstream sequence: 5′-AACAUUCAACCUGUCGGUGAGU-3′, and downstream sequence: 5′-UCACCGACAGGUUGAAUGUUUU-3′. Luciferase activity was determined 48 h post-transfection, using the Dual-Luciferase Reporter assay kit (Promega, USA) and measured by using a TD 20/20n luminometer (Turner Biosystems, USA). These data represented the average of three independent experiments, performed in three wells each time, and were shown with the standard error.

### Real-Time Quantitative PCR

Total RNA was isolated from Neuro-2a cells or mouse hippocampus by TRIzol reagent (Invitrogen, USA). The RNA was subjected to reverse transcription using the HiScript II 1st Strand cDNA Synthesis Kit (+gDNA wiper) (Vazyme Biotech, China). Real-time PCR analysis was performed using the following primers (Ribo Life Science, China): H-2D^b^ (forward primer: 5′-GGCGAGTGCGTGGAGTG-3′; reverse primer: 5′-CATCACAAAAGCCACCACAGC-3′), H-2K^b^ (forward primer: 5′-GACGAGAGACTCAGGGCCTAC-3′; reverse primer: 5′-AACGGTCGCCATGTTGGAGAC-3′), GAPDH (forward primer: 5′-AGGTCGGTGTGAACGGATTTG-3′; reverse primer: 5′-TGTAGACCATGTAGTTGAGGTCA-3′), miR34a (forward primer: 5′-TGGCAGTGTCTTAGCTGGTTGT; reverse primer: 5′-CGAATTCTAGAGCTCGAGGCAG), U6 (forward primer: 5′-CGCAAATTCGTGAAGCGTTCC; reverse primer: 5′-CGAATTCTAGAGCTCGAGGCAG). Relative quantification was performed following the instruction for ChamQ SYBR qPCR Master Mix (Vazyme Biotech, China) with a Real-Time PCR system (Applied Biosystems, USA). U6 snRNA was used as the endogenous control for miRNA Real-time PCR analyses, GAPDH was used as a housekeeping gene for normalization. All reactions were run in triplicate. Results were represented as fold expression of the control.

### Western Blotting Analysis

The whole-cell lysates were lysed by RIPA lysis buffer (Sangon Biotech, China) with protease inhibitors. Protein extracted from cells was quantified by the BCA protein assay kit (Thermo, USA). Equal amounts of protein (35–45 μg) in a protein sample buffer were denatured at 100°C for 10 min. The protein samples were loaded into the SDS-PAGE gel sample well. To observe the electrophoresis process, and to determine the molecular weight of proteins, pre-stained protein ladders were added into the gel as well. Proteins were transferred to a polyvinylidene fluoride (PVDF) membrane (Millipore, USA) after SDS-PAGE was finished. PVDF membrane was incubated in 5% BSA (AMRESCO, USA) in Tris-buffered saline at 37°C for 2 h to block nonspecific binding, and then probed with primary antibodies at 4°C for 12–16 h (anti-GAPDH, 1:1,000, Cell Signaling, USA; anti-classical MHC-I, 1:1,000, Bioworld, USA; anti-Flag, 1:2,000, Bioworld, China). After washed with TBST, the membrane was incubated with a peroxidase-conjugated secondary antibody for 1.5 h at room temperature. The Immunodetection of the PVDF membrane was performed with an enhanced chemiluminescent (ECL) substrate (Pierce, USA). Detection was performed using a Luminoimage analyzer (Tanon, China) and quantification was analyzed by densitometry using ImageJ software (USA).

### Immunofluorescence Staining

Mouse anti-microtubule-associated protein2 (MAP2, 1:200, Abcam, USA), Flag (1:100, Bioworld, China), DAPI (1:1,000, Sigma–Aldrich, USA), and secondary antibodies conjugated with Alexa Fluor 546 (1:500, Invitrogen, USA) were used in immunofluorescence experiments. Coverslips were rinsed in PBS to remove medium before fixed by 4% paraformaldehyde (Sigma Aldrich, USA) in 0.01 M PBS for 20 min. Then coverslips were rinsed three times in PBS. Cells were permeabilized with 0.25% Triton X-100 (Sigma Aldrich, USA) for 15 min and washed three times again. Cells were blocked with BSA for 2 h before incubated with primary antibodies overnight at 4°C in a humid chamber. After that, cells were rinsed and incubated with secondary antibodies for 2 h at room temperature in the dark. After washed three times, the coverslips were sealed with glycerin and observed with a fluorescence microscope (Olympus Fluoview FV 1000, Japan).

### Sholl Analysis

Sholl Analysis is a method for quantitative analysis of neuron axons and dendrites. For the Sholl analysis, a group of concentric circles is superimposed on the cell body and the branch patterns of neuron dendrites and axons in different regions are obtained by calculating the number of branches that intersect each circle, thereby quantitatively characterizing the imaged nerve metamorphic characteristics.

### Statistical Analysis

All *in vitro* experiments were repeated at least three times. All statistical analyses were performed with one-way ANOVA or Student’s *t*-test using GraphPad Prism 8.0.1 (GraphPad Software, USA). The numerical data are expressed as the mean ± standard error of the mean (SEM). A value of *P* < 0.05 was considered to be statistically significant. No data points were excluded.

## Results

### MiR34a Targets MHC-I Molecules

**Figure 1:**
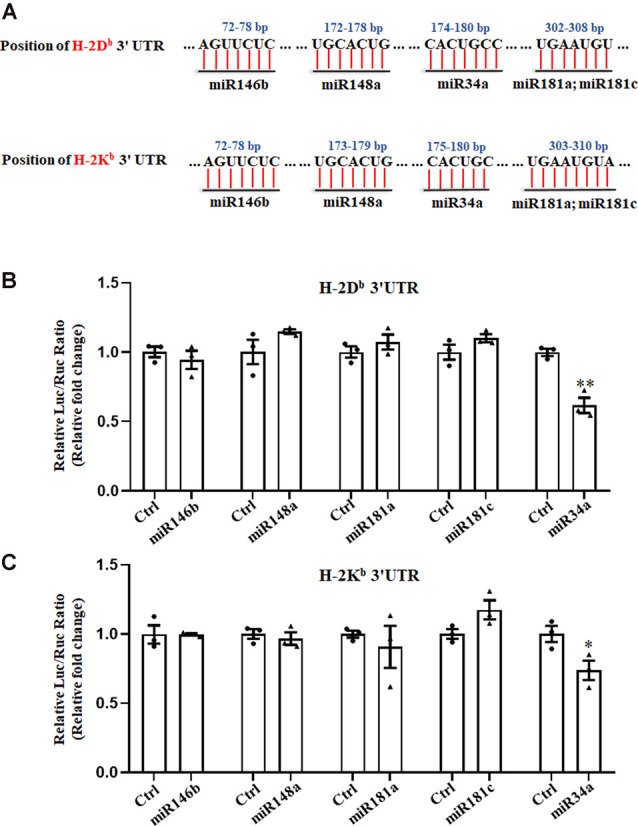
Searching for miRNAs that target H-2K^b^ and H-2D^b^. **(A)** A schematic drawing of the H-2K^b^ and H-2D^b^ 3′UTR with indication of miR146b, miR148a, miR34a, miR181a and miR181c putative binding sites within the 3′UTR. **(B,C)** HEK293T cells were cotransfected with a dual luciferase reporter containing the wild-type H-2D^b^ 3′UTR **(B)** or H-2K^b^ 3′UTR **(C)** and different miRNA mimics with their controls for 48 h. All data are presented as the means ± SEM of three individual experiments. Values indicated the Firefly luciferase activity normalized to Renilla luciferase activity. *P < 0.05; **P < 0.01 vs. negative control.

**Figure 2:**
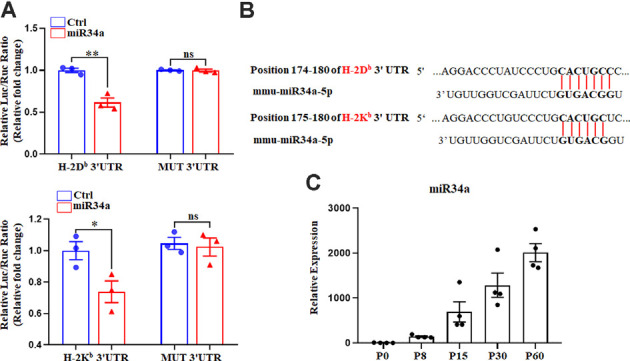
MiR34a participates in the regulation of H-2D^b^ and H-2K^b^. **(A)** HEK293T cells were cotransfected with a dual luciferase reporter containing the wild-type or mutant constructs of H-2D^b^ 3′ UTR (upper panel) or H-2K^b^ 3′ UTR (lower panel) for 48 h. All data are presented as the means ± SEM of three individual experiments. Values indicated the Firefly luciferase activity normalized to Renilla luciferase activity. *P < 0.05; **P < 0.01 vs. negative control; ns: non-significant. **(B)** Putative miR34a binding sites in the 3′ UTR of H-2K^b^ and H-2D^b^, The potential complementary residues are shown in bold. **(C)** miR34a expression in different periods of mice hippocampus. Data are presented as the means ± SEM of four individual experiments.

We previously reported on the expression of H-2K^b^ and H-2D^b^ mRNA and protein from P0 to P60 in the hippocampus of C57BL/6 mice (Li et al., 2020). There was an increase of H-2K^b^ and H-2D^b^ proteins from P0 to P15 before it decreased to a relatively low level, despite a sustained increased H-2K^b^ and H-2D^b^ mRNA from P0 to P60, suggesting post-transcriptional regulation mechanisms such as miRNAs might be involved after P15. According to TargetScan and miRanda analysis, several miRNAs such as miR146b, miR148a, miR181a, miR181c, and miR34a were predicted to target H-2K^b^ and H-2D^b^ (Figure 1A). To confirm if H-2D^b^ and H-2K^b^ expression was regulated by those miRNAs, the wild type sequence of H-2D^b^ or H-2K^b^ 3′UTR was cloned into pmirGLO dual-luciferase reporter vectors and co-transfected with miRNA mimics in HEK293T cells. MicroRNA mimics were chemically synthesized to enhance the function of endogenous miRNAs. Of all the miRNAs investigated, only miR34a mimics manifested attenuated luciferase signal compared to the non-targeting Ctrl (Figures 1B,C). This downregulation was aborted when transfected with mutated plasmids and miR34a mimics (Figure 2A, upper panel). The mutated sequence was designed according to TargetScan where the estimated binding site of miR34a to H-2D^b^ or H-2K^b^ was changed (Figure 2B). The same effect was also shown in H-2K^b^ 3′UTR-luciferase plasmid with miR34a mimics (Figure 2A, lower panel). In summary, these results showed that the binding of miR34a to MHC-I mRNA was dependent on the 3′UTR sequence of H-2D^b^ or H-2K^b^ and miR34a could decrease MHC-I expression.

It is worth noting that miR34a was expressed over different periods of C57BL/6 mouse hippocampal development. MiR34a was expressed at a low level postnatally, while expression increased dramatically after P15 (Figure 2C). Since H-2K^b^ and H-2D^b^ proteins were dramatically decreased after P15 in the hippocampus, miR34a might contribute to the regulation of MHC-I expression during this developmental stage.

### MiR34a Regulates MHC-I Expression in Neuro-2a Cell Lines

**Figure 3:**
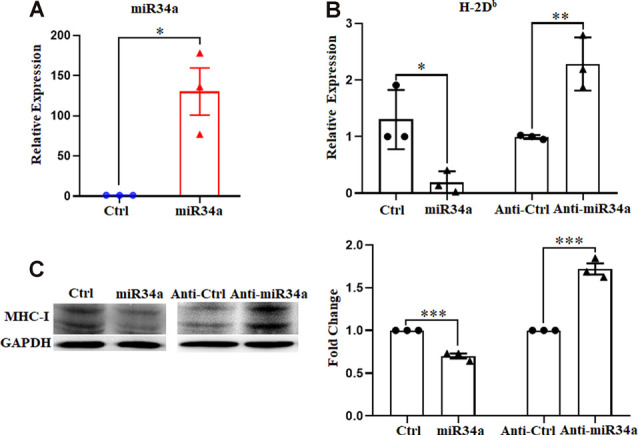
MiR34a has a negative impact on classical MHC-I expression in Neuro-2a cells. **(A)** Increased expression of miRNA-34a in neuro-2a cells transfected with miR34a mimic for 24 h was detected by RT-qPCR. *P < 0.05 vs. negative control. **(B)** Neuro-2a cells were transfected with 50 nM miR34a mimic or 100 nM miR34a inhibitor for 24 h followed by measurement of H-2D^b^ expression with RT-qPCR analysis. *P < 0.05; **P < 0.01 vs. negative control. **(C)** Expression of the classical MHC-I protein in neuro-2a cells transfected with miR control, miR34a mimic, inhibitor control or miR34a inhibitor for 48 h was detected by western blotting. GAPDH was probed as an internal control for experiments. Densitometric analysis of the classical MHC-I expression using ImageJ is presented. ***P < 0.001 vs. negative control. All data are presented as the means ± SEM of three independent experiments.

To further verify the regulation of MHC-I by miR34a, we investigated its effect on Neuro-2a cell lines using miR34a mimics and inhibitors. Neuro-2a cells were previously reported to express H-2D^b^ but not H-2K^b^ (Li et al., 2020), so only the expression of H-2D^b^ was investigated here. In contrast to miRNA mimics, the miRNA inhibitors could effectively inhibit mature miRNA function without degrading it (the endogenous miRNA levels remained unchanged). After transfecting miRNA mimics into Neuro-2a cells, miR34a expression was significantly upregulated as examined by real-time PCR (Figure 3A). As shown in Figure 3B, overexpressing miR34a mimics alleviated H-2D^b^ expression in Neuro-2a cells. At the same time, the transfection of miR34a inhibitors increased H-2D^b^ expression. Consistent with these findings, the change of MHC-I protein was identical with MHC-I mRNA when treated with miR34a mimics or inhibitors (Figure 3C). Taken together, these results suggested that the expression of MHC-I mRNA and proteins was regulated by miR34a in Neuro-2a cell lines.

### Manipulated miR34a Expression Changes MHC-I Levels in Primary Mouse Hippocampal Neurons

**Figure 4:**
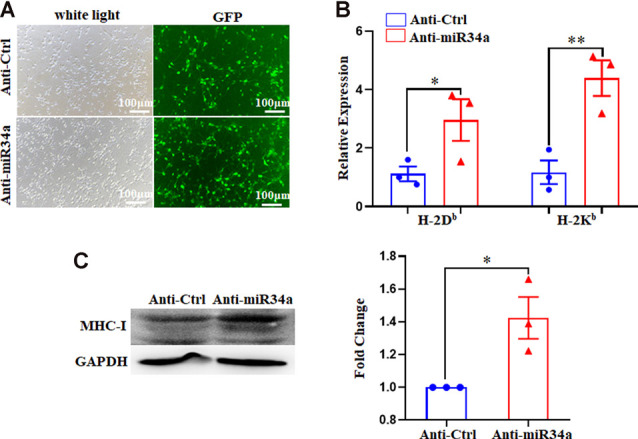
MiR34a blockade induces classical MHC-I expression in primary hippocampal neurons. **(A)** Representative images of transfection efficiency with anti-Ctrl-GFP lentivirus or anti-miR34a-GFP lentivirus infection in primary hippocampal neurons. Cells were infected with lentivirus at DIV 1 and observed at DIV 5 with a fluorescence microscope. Bars: 100 μm. **(B)** Increased levels of H-2D^b^ and H-2K^b^ in primary hippocampal neurons infected with anti-miR34a-GFP lentivirus for 4 days compared to those infected with anti-Ctrl-GFP lentivirus. The level of indicated mRNA was determined by RT-qPCR. *P < 0.05; **P < 0.01 vs. negative control. **(C)** Expression of the classical MHC-I protein in mouse primary hippocampal neurons infected with anti-Ctrl-GFP lentivirus or anti-miR34a-GFP lentivirus for 4 days as determined by western blotting. Densitometric data of the classical MHC-I protein expression using ImageJ are presented as the means ± SEM of three independent experiments. *P < 0.05 vs. negative control. All data are presented as the means ± SEM of three independent experiments.

**Figure 5:**
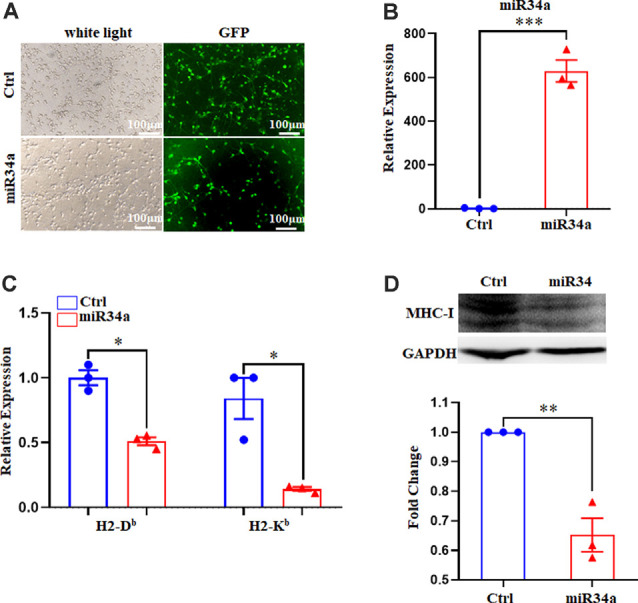
Increased miR34a inhibits classical MHC-I expression in primary hippocampal neurons. **(A)** Representative images of transfection efficiency with Ctrl-GFP lentivirus or miR34a-GFP lentivirus infection in primary hippocampal neurons. Cells were infected with lentivirus at DIV 1 and observed at DIV 5 with a fluorescence microscope. Bars: 100 μm. **(B)** Cells were assessed for miRNA-34a expression at the mRNA levels after lentivirus infection. ***P < 0.001 vs. negative control. **(C)** Decreased H-2D^b^ and H-2K^b^ in primary hippocampal neurons infected with miR34a-GFP lentivirus compared to those infected with Ctrl-GFP lentivirus for 4 days. The level of indicated mRNA was determined by RT-qPCR. *P < 0.05 vs. negative control. **(D)** Expression of the classical MHC-I protein in mouse primary hippocampal neurons infected with Ctrl-GFP lentivirus or miR34a-GFP lentivirus for 4 days as determined by western blotting. Densitometric data of the classical MHC-I protein expression using ImageJ are presented as the means ± SEM of three independent experiments. **P < 0.01 vs. negative control. All data are presented as the means ± SEM of three independent experiments.

Since Neuro-2a is a murine neuroblastoma cell line, whether regulation of MHC-I by miR34a also applied to neurons required additional investigation. We dissected primary hippocampal neurons from embryonic E16.5–18.5 C57BL/6 mice. The cultured neurons were infected with anti-miR34a-GFP lentivirus which expressed miR34a specific sponge at DIV (days *in vitro*) 1. The sponge contains a series of antisense sequences of miR34a that can bind to miR34a through complementary base pairing, thereby preventing miR34a from binding to its target sequence. Transfection efficiency was estimated at approximately 80% after 4 days post lentivirus infection as observed by fluorescence microscopy (Figure 4A). As compared to neurons infected with a control virus, both H-2D^b^ and H-2K^b^ mRNA and protein expression were upregulated in neurons infected with anti-miR34a-GFP lentivirus (Figures 4B,C). In concordance with this, infection of miR34a-GFP lentivirus (Figure 5A), which increased the endogenous level of miR34a (Figure 5B), inhibited both MHC-I mRNA and protein expression in cultured hippocampal neurons (Figures 5C,D). In general, we confirmed that MHC-I expression was regulated by miR34a in primary cultured mouse hippocampal neurons.

### Dendritic Growth and Branching Varies According to Changes of miR34a Expression in Hippocampal Neurons, Which is Mediated by MHC-I Molecules

**Figure 6:**
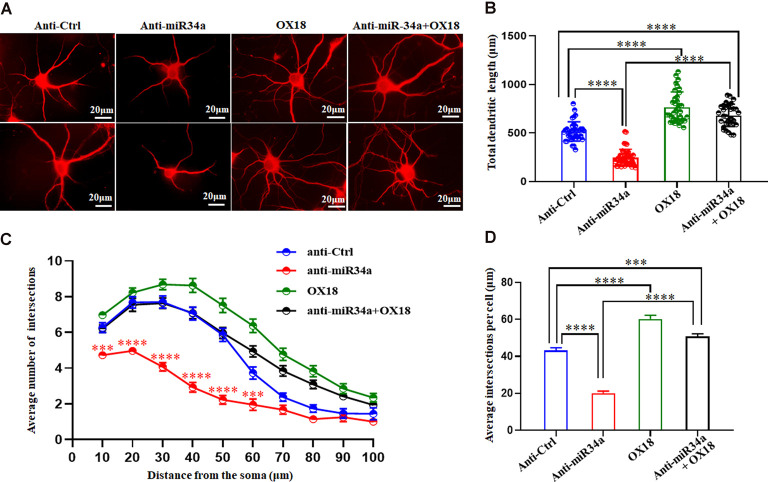
MiR34a deficiency in hippocampal neurons decreases dendritic growth and branching. **(A)** Primary hippocampal neurons infected with anti-Ctrl-GFP lentivirus or anti-miR34a-GFP lentivirus were fixed and immunostained for MAP2 at DIV 5 to confirm shape of neurites. MHC class I antibody OX18 was added into the culture medium at DIV 3 to block MHC class I expression. A combination of anti-miR34a-GFP lentivirus treatment and antibody blockade was applied to neurons and it’s morphology was monitored by MAP2 at DIV 5. Bars: 20 μm. **(B)** Quantification of neurite length. ****P < 0.0001 vs. negative control. **(C,D)** Sholl analysis was performed on these neurons. Sholl profile and the average total numbers of intersections per cell were shown (*n* = 37 anti-Ctrl group, *n* = 43 anti-miR34a group, *n* = 36 OX18 blockade group, *n* = 33 anti-miR34a + OX18 group, ***P < 0.001; ****P < 0.0001).

One of the established roles of MHC-I in neuronal development is to inhibit axon and dendrite outgrowth in hippocampal and cortical neurons both *in vitro* and *in vivo* (Washburn et al., 2011). We explored whether changes in the expression of MHC-I by miR34a would affect the shape of neurites in cultured hippocampal neurons. Neurons were infected with anti-miR34a-GFP lentivirus or anti-Ctrl-GFP lentivirus at DIV 1. After cultured for several days, they were fixed and immunostained for MAP2 at DIV 5. Compared to the anti-Ctrl-treated neurons, a significant decrease of total dendrite length in anti-miR34a-GFP-treated neurons was observed (Figures 6A,B). We also detected a dramatic decrease in dendritic complexity in anti-miR34a-GFP-treated neurons (Figures 6C,D) as revealed by Sholl analysis. To confirm that this effect is mediated by increased MHC-I expression upon anti-miR34a-GFP lentivirus infection, blockade of MHC-I was achieved by adding a monoclonal MHC class I antibody OX18 to the culture medium at DIV 3 and neurons was fixed and immunostained for MAP2 at DIV 5. Neurons treated with the MHC class I antibody exhibited a marked increase in dendritic length and complexity, and the antibody successfully rescued decreased neurite outgrowth of anti-miR34a-GFP-treated neurons (Figures 6A–D).

**Figure 7:**
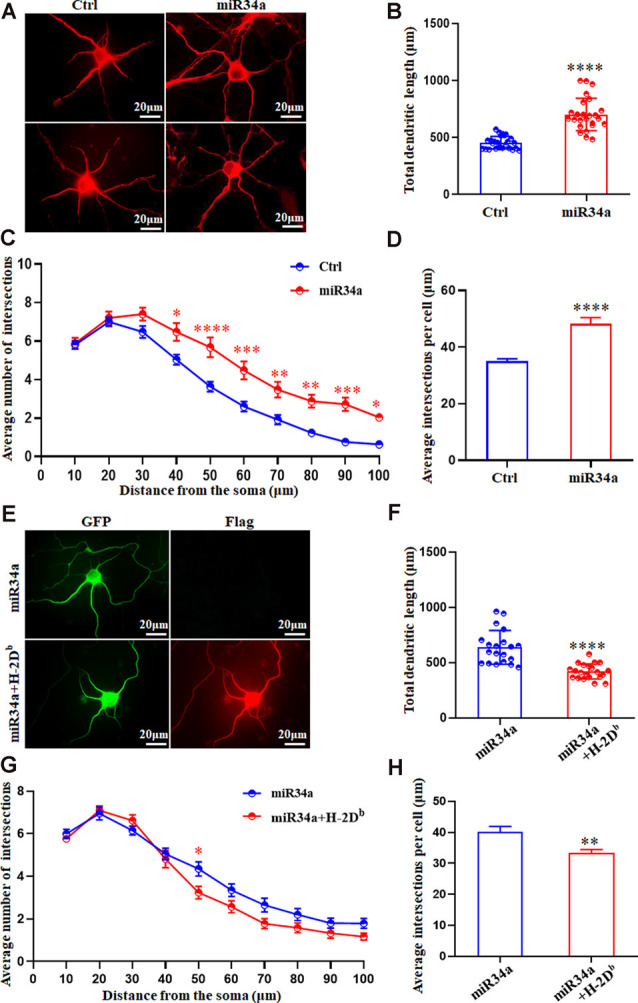
MiR34a overexpression in hippocampal neurons increases dendritic growth and branching. **(A)**Primary hippocampal neurons were infected with miR34a-GFP lentivirus for 4 days. Neurons were fixed and immunostained for MAP2 at DIV 5. Bars: 20 μm. **(B)** Quantification of neurite length. ****P < 0.0001 vs. negative control. **(C,D)** Sholl analysis was performed on these neurons. Sholl profiles and the average total numbers of intersections per cell were shown (*n* = 25 Ctrl group, *n* = 25 miR34a group. *P < 0.05; **P < 0.01; ***P < 0.001; ****P < 0.0001 vs. negative control). **(E)** Primary hippocampal neurons were infected with miR34a-GFP lentivirus with/without H-2D^b^-Flag-lentivirus at DIV 1. Neurons were immunostained by anti-Flag antibody at DIV 5. The expression of GFP and Flag was observed with a fluorescence microscope. Bars: 20 μm. **(F)** Quantification of neurite length was shown. ****P < 0.0001 vs. miR34a-GFP. **(G,H)** Sholl analysis was performed on these neurons. Sholl profiles and the average total numbers of intersections per cell were shown (*n* = 20 miR34a group, *n* = 21 miR34a + H-2D^b^ group. *P < 0.05; **P < 0.01 vs. miR34a-GFP).

In concordance with this, miR34a-GFP lentivirus treated neurons showed dramatically increased total dendritic length compared to Ctrl-GFP neurons (Figures 7A,B), as well as dendritic branching (Figures 7C,D). Increased expression of H-2D^b^ in HEK293T cells and cultured hippocampus neurons was achieved by infection with an H-2D^b^-Flag-lentivirus for 96 h and stained with Flag antibody (Supplementary Figure 1; Figure 7E). In our previous research, overexpression of H-2D^b^ in neurons induced enhanced expression of MHC class I molecules could decrease neurite growth and branching (Shen et al., 2019). We further demonstrated miR34a-mediated enhancement of total dendritic length was thoroughly blocked by overexpression of H-2D^b^ (Figures 7E,F), and miR34a-mediated increment of the average number of intersections was slightly reduced by overexpression of H-2D^b^ in hippocampus neurons (Figures 7G,H). This phenomenon indicated that miR34a shaped neurite neuron morphology in cultured hippocampal neurons by modulation of MHC-I molecules.

## Discussion

In the immune system, MHC-I molecules are constitutively expressed on almost all nucleated cells, binding and presenting peptides from processed cellular antigens to cytotoxic T cells. Apart from that, MHC-I proteins can be induced upon cytokine stimulation. Both constitutive and inducible expression of MHC-I molecules is mainly dependent on the binding of transcriptional factors to conserved promoter elements: enhancer A (EnhA), IFN-stimulated response element (ISRE), and the SXY module in the MHC-I promoter. NLRC5 cooperates with transcription factors that bind to the SXY module to drive MHC-I expression (Meissner et al., 2012; Neerincx et al., 2013).

Since people find MHC-I molecules are expressed throughout the healthy CNS, accumulating evidence validates their critical roles in neural development. They regulate activity-dependent refinement of synaptic plasticity (Huh et al., 2000; Glynn et al., 2011; Datwani et al., 2009; Lee et al., 2014), limit the establishment of synaptic connections and the degree of dendrite arborization (Bilousova et al., 2012; Shen et al., 2019), as well as inhibit NMDAR function and hippocampal-dependent memory formation (Fourgeaud et al., 2010; Wu et al., 2011). Even though regulation of MHC-I expression has been extensively investigated in the immune system for many years now, a multitude of basic questions about the mechanism(s) governing neuronal MHC-I expression remains poorly understood. We previously reported that calcium-dependent protein kinase C (PKC) was important in relaying intracranial kainic acid (KA) stimulation signals to up-regulate MHC-I expression in hippocampal neurons and that this signaling cascade relied on activation of the MAPK pathway (Lv et al., 2015). We also confirmed that NLRC5, but not CIITA, was broadly expressed in the mouse brain and promoted MHC-I expression in hippocampal neurons (Li et al., 2020). In this study, we analyze the regulation of MHC-I expression from a new perspective: regulation by miRNAs in hippocampal neurons, adding new information regarding the tight and complex regulatory system that controls classical MHC-I molecule levels during the development of the hippocampus.

MicroRNAs are endogenously expressed non-coding RNAs that regulate complementary mRNA expression. In vertebrates, microRNAs are ubiquitously expressed in the brain with a growing body of evidence showing their functions in neural development and their contributions to neurodevelopmental diseases (Fineberg et al., 2009). To exert their regulatory function, miRNAs silence gene expression at the post-transcription level by guiding Argonaute (AGO) family proteins to constitute miRNA-induced silencing complexes (miRISCs), finally degrading gene transcripts if mRNA is fully complementary to the miRNA seed region (Jonas and Izaurralde, 2015). In this way, the sequence of mRNAs can be used for the prediction of possible binding miRNAs by matching their sequence with the seed region of miRNA. To begin our work, computational algorithms identify several candidate miRNAs by searching for the presence of conserved miRNA seed sequences that match H-2K^b^ and H-2D^b^ mRNA. MiR146b, miR148a, miR181a, miR181c, and miR34a are further tested by luciferase activity assay in HEK293T cells for their ability to regulate H-2K^b^ and H-2D^b^ expression. MiR34a, which is perfectly complementary to the seed sequence of H-2D^b^ and partially complementary to the seed sequence of H-2K^b^, is the only miRNA among all candidates that effectively decrease H-2K^b^ and H-2D^b^ 3′UTR-luciferase activity in HEK293T cells. In cases when the mRNA target is only partially complementary to the miRNA, additional protein partners are recruited by AGO proteins to mediate mRNA silencing in the absence of direct cleavage by AGO protein. Other post-transcriptional regulation mechanisms such as translation inhibition can also be used by miRNA to regulate protein expression of the target gene. In our study, manipulating miR34a expression can change both H-2K^b^ mRNA level and MHC-I protein expression in neuro-2a cells and cultured mouse hippocampal neurons, implying that both translational repression and mRNA degradation contribute to miR34a regulated MHC-I expression. Further experiments are required to dissect the detailed pathway(s) used by miR34a to regulate MHC-I expression in hippocampal neurons.

Although we confirm the regulation of MHC-I expression by miR34a in cultured hippocampal neurons, whether it can repress MHC-I expression in the developing hippocampus *in vivo* is still unknown. So far, miR34a is shown to be expressed in the CNS. It influences terminal neuronal differentiation, regulates neurite outgrowth, and mediates dendritic spine morphology and function (Agostini et al., 2011a,b). As a brain-enriched microRNA, miR34a upregulates with brain age (Li et al., 2011; Jauhari et al., 2018). It can modulate mitochondrial activity by regulating mitochondrial protein abundance in aging cells, eventually leading to age-related diseases (Rippo et al., 2014). During the development of the mouse hippocampus, it was reported that P15 was a critical period for refinement of neuronal connections (Hashimoto and Kano, 2003) when MHC-I is expressed. Inappropriate neuronal MHC-I expression after this critical period in the hippocampus could be deleterious, thus shutting down MHC-I expression after p15 is necessary. We previously demonstrated that the MHC-I protein level was dramatically decreased after P15 while MHC-I mRNA was kept at in high level (Li et al., 2020). In this study, we find that miR34a is expressed at a low level in early postnatal mice, while it increases dramatically after P15, following the peak of MHC-I protein expression at P15. As we find that miR34a can decrease MHC-I expression in primary cultured hippocampus neurons, expression of miR34a at this stage could be one of the mechanisms for decreasing MHC-I expression. However, our *in vitro* experiment shows decreased both MHC-I mRNA and protein expression following increased miR34a, which is not in concordance with *in vivo* findings that only MHC-I protein is decreased. Other transcriptional and post-transcriptional regulation mechanisms may contribute to divergent expression of MHC-I mRNA and protein expression after P15. Thus, the regulation of MHC-I by miR34a in the hippocampal neuron *in vivo* still needs to be investigated.

Our findings also indicate that decreased miR34a inhibits neurite outgrowth and branching in cultured hippocampal neurons, which recapitulates our previous finding that upregulated MHC-I expression can decrease neurite outgrowth (Shen et al., 2019). We also confirm the causal relationship between miR34a mediated modulation of MHC-I and hippocampal dendritic growth by successfully rescuing the decreased length and branching of anti-miR34a-GFP lentivirus treated neurons by blocking with MHC class I antibody. On the other hand, the miR34a-GFP lentivirus-mediated increase of dendritic complexity can be blocked by overexpression of H-2D^b^. However, a study from Agostini et al. (2011b) reports that miR34a negatively affects dendritic outgrowth of cortical and hippocampus neurons, which is different from our results. There are several possible reasons for this discrepancy. First, in their report, hippocampal neurons are co-transfected with a GFP expression and a miR34a overexpression plasmids (PremiR34a) with siPORT NeoFX transfection agent. While in our study, primary mouse hippocampal neurons are infected with lentivirus to inhibit or overexpress miR34a expression. With high transfection efficiency shown by immunofluorescence in neurons, we can confirm the expression of miR34a after virus infection by Real-time quantitative PCR. We find that inhibition of miR34a decreases dendritic growth and branching and at the same time, overexpression of miR34a increases dendritic growth and branching in cultured hippocampal neurons. Secondary, they transfect neurons at DIV 4, and cells are analyzed 72 h later while in our study neurons are infected at DIV 1 and analyzed 96 h later. We suppose that different transfection methods and different time points to treat the neurons cause inconsistent results in neuron morphology. In conclusion, all these results will help us to better understand the tight regulation of MHC-I expression in the developing hippocampus, which is also helpful towards elucidating its role in aging and degenerative diseases.

## Data Availability Statement

All datasets presented in this study are included in the article/Supplementary Material.

## Ethics Statement

The animal study was reviewed and approved by Institutional Animal Care and Use Committee (IACUC) of the Medical School of Southeast University (approval ID: SYXK-2010.4987).

## Author Contributions

YH conceived of the presented idea and carried out the experiments, YH wrote the manuscript with support from YS and PL. All authors discussed the results and contributed to the final manuscript. JZ helped supervise the project. All authors contributed to the article and approved the submitted version.

## Conflict of Interest

The authors declare that the research was conducted in the absence of any commercial or financial relationships that could be construed as a potential conflict of interest.

## Supplementary Material

SUPPLEMENTARY FIGURE 1 Overexpression of H-2D^b^ in HEK293T cells was achieved using lentivirus delivery. Cell lysates were immunoblotted with Flag and GAPDH antibodies to confirm the expression of H-2D^b^.

SUPPLEMENTARY TABLE 1 Sequences of H-2D^b^ and H-2K^b^ 3′UTR are listed, and lentivirus-mediated gene sequences are shown.
