# Supplementary figures and images for: MiR34a Regulates Neuronal MHC Class I Molecules and Promotes Primary Hippocampal Neuron Dendritic Growth and Branching
Source: Front Cell Neurosci. 2020 Oct 28;14:573208. doi: 10.3389/fncel.2020.573208 (PMC7655649; doi:10.3389/fncel.2020.573208)

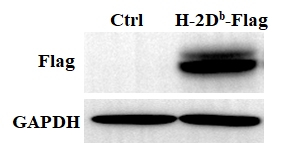

Supplement: SUPPLEMENTARY FIGURE 1 — Overexpression of H-2Db in HEK293T cells was achieved using lentivirus delivery. Cell lysates were immunoblotted with Flag and GAPDH antibodies to confirm the expression of H-2Db. [file Image_1.TIF]
