# Supplementary material for: MiR34a Regulates Neuronal MHC Class I Molecules and Promotes Primary Hippocampal Neuron Dendritic Growth and Branching
Source: Front Cell Neurosci. 2020 Oct 28;14:573208. doi: 10.3389/fncel.2020.573208 (PMC7655649; doi:10.3389/fncel.2020.573208)
Supplement: SUPPLEMENTARY TABLE 1 — Sequences of H-2Db and H-2Kb 3′UTR are listed, and lentivirus-mediated gene sequences are shown. [file Table_1.DOCX]

**Supplementary Material and Methods**

**The length of H-2D^b^ 3’UTR is 627bp. Sequences are as follows:**

AGACAGCTGCCTGGACTGTACTGAGTGACAGACGATGTGTTCAGGTCTCTCCTGTGACATCCAGAGCCCTCAGTTCTCTTTACACAACATTGTCTGATGTTCCCTGTGAGCTTGGGTTCAGTGTGAAGAACTGTGGAGCCCAGCCTGCCCTGCACACCAGGACCCTATCCCTGCACTGCCCTGTGTTCCCTTCCATAGCCAACCTTGCTGCTCCAGCCAAACACTGGGGGACATCTGCATCCTGTAAGCTCCATGCTACCCTGAGCTGCAGCTCCTCACTTCCACACTGAGAATAATAATTTGAATGTGGGTGGCTGGAGAGATGGCTCAGCGCTGACTGCTCTTCCAAAGGTCCTGAGTTCAAATCCCAGCAACCACATGGTGGCTCACAACCATCTGTAATGGGATCTAACACCCTCTTCTGCAGTGTCTGAAGACAGCTACAGTGTACTTACATATAATAATAAATAAGTCTTTAAAAAATAATTTGAAAGTGACCTTGATTGTTAACATCTTGATCTAGGGCTGATTTCTTGTTAATTTCATGGATTGAGAATGCTTAGAGGTTTTTTTGTTTGTTTGATTGATTTTTTTGAAGAAATAAATGGCAGATGAAGGAACTTCCAG

**The length of H-2K^b^ 3’UTR is 483bp. Sequences are as follows:**

AGACAGCTGCCTGGAGTGGACTTGGTGACAGACAATGTCTTCTCATATCTCCTGTGACATCCAGAGCCCTCAGTTCTCTTTAGTCAAGTGTCTGATGTTCCCTGTGAGCCTATGGACTCAATGTGAAGAACTGTGGAGCCCAGTCCACCCCTCTACACCAGGACCCTGTCCCTGCACTGCTCTGTCTTCCCTTCCACAGCCAACCTTGCTGGTTCAGCCAAACACTGAGGGACATCTGTAGCCTGTCAGCTCCATGCTACCCTGACCTGCAACTCCTCACTTCCACACTGAGAATAATAATTTGAATGTAACCTTGATTGTTATCATCTTGACCTAGGGCTGATTTCTTGTTAATTTCATGGATTGAGAATGCTTAGAGGTTTTGTTTGTTTGTTTGATTGATTTGTTTTTTTGAAGAAATAAATGATAGATGAATAAACTTCCAGAATCTGGGTCACTAAAAAAAAAAAAAAAAAAAAAAAA

**The lentivirus-mediated gene sequences are as follows:**

**Ctrl sequence:**

TTCTCCGAACGTGTCACGT

**Anti-miR-34a sequence:**

ACAACCAGCTAAGACACTGCCA

**miR-34a sequence:**

TGCTGGAGGAGTGTGTCATACCTCGGTAGGGTCCACTACACATCTTTCTCCCGCAGCCTCTCCATCTTCCTGTGACTGCGGGCGCCTCAGCCTGGGCTGGCCAGCTGTGAGTAATTCTTTGGCAGTGTCTTAGCTGGTTGTTGTGAGTATTAGCTAAGGAAGCAATCAGCAAGTATACTGCCCTAGAAGTGCTGCACATTGTTGGGCCGAGAAGGAAAAGGTCAGAGGTCAGCAACGCCCACACCCCTGAGAGGCGCTGGACTTGCGGAGCTGCTCGACCATACTGGTGGGTATGGGATGCTTTTTT

**H-2D^b^ CDS sequence:**

ATGGGGGCGATGGCTCCGCGCACGCTGCTCCTGCTGCTGGCGGCCGCCCTGGCCCCGACTCAGACCCGCGCGGGCCCACACTCGATGCGGTATTTCGAGACCGCCGTGTCCCGGCCCGGCCTCGAGGAGCCCCGGTACATCTCTGTCGGCTATGTGGACAACAAGGAGTTCGTGCGCTTCGACAGCGACGCGGAGAATCCGAGATATGAGCCGCGGGCGCCGTGGATGGAGCAGGAGGGGCCGGAGTATTGGGAGCGGGAAACACAGAAAGCCAAGGGCCAAGAGCAGTGGTTCCGAGTGAGCCTGAGGAACCTGCTCGGCTACTACAACCAGAGCGCGGGCGGCTCTCACACACTCCAGCAGATGTCTGGCTGTGACTTGGGGTCGGACTGGCGCCTCCTCCGCGGGTACCTGCAGTTCGCCTATGAAGGCCGCGATTACATCGCCCTGAACGAAGACCTGAAAACGTGGACGGCGGCGGACATGGCGGCGCAGATCACCCGACGCAAGTGGGAGCAGAGTGGTGCTGCAGAGCATTACAAGGCCTACCTGGAGGGCGAGTGCGTGGAGTGGCTCCACAGATACCTGAAGAACGGGAACGCGACGCTGCTGCGCACAGATTCCCCAAAGGCACATGTGACCCATCACCCCAGATCTAAAGGTGAAGTCACCCTGAGGTGCTGGGCCCTGGGCTTCTACCCTGCTGACATCACCCTGACCTGGCAGTTGAATGGGGAGGAGCTGACCCAGGACATGGAGCTTGTGGAGACCAGGCCTGCAGGGGATGGAACCTTCCAGAAGTGGGCATCTGTGGTGGTGCCTCTTGGGAAGGAGCAGAATTACACATGCCGTGTGTACCATGAGGGGCTGCCTGAGCCCCTCACCCTGAGATGGGAGCCTCCTCCGTCCACTGACTCTTACATGGTGATCGTTGCTGTTCTGGGTGTCCTTGGAGCTATGGCCATCATTGGAGCTGTGGTGGCTTTTGTGATGAAGAGAAGGAGAAACACAGGTGGAAAAGGAGGGGACTATGCTCTGGCTCCAGGCTCCCAGAGCTCTGAAATGTCTCTCCGAGATTGTAAAGCG
